# ANZAED 2021 Hybrid Conference: Oral and Poster Abstracts

**DOI:** 10.1186/s40337-021-00505-6

**Published:** 2021-12-22

## A.1

### Emotion dysregulation across the span of eating disorders: Findings from a community sample of adolescents

#### Nora Trompeter^1^, Kay Bussey^1^, Miriam Forbes^1^, Jonathan Mond^2^, Phillipa Hay^3^, Mitchell Cunningham^4^, Deborah Mitchison^3^

##### ^1^Macquarie University, New South Wales, Australia. ^2^University of Tasmania, Tasmania, Australia. ^3^Western Sydney University, New South Wales, Australia. ^4^University of Sydney, New South Wales, Australia

**Correspondence**: Nora Trompeter (nora.trompeter@mq.edu.au)

*Journal of Eating Disorders* 2021, **9(1)**: A.1

Emotion dysregulation is a key factor within eating pathology. However, less is known about specific emotion regulation difficulties experienced by adolescents with eating disorders. The present study examined differences in emotion dysregulation between eating disorder diagnostic groups and the relationship between eating disorder behaviors and specific facets of emotion dysregulation. Participants were 2783 adolescents, 11–19 years (M = 14 years, 9 months, SD = 1 year, 6 months), who completed self-report measures. Adolescents were identified as not having an eating disorder (n = 2266) or meeting criteria for a specific eating disorder: anorexia nervosa or atypical anorexia nervosa (n = 37), bulimia nervosa (n = 136), other specified feeding or eating disorder characterised by binge eating or purging (n = 314), and unspecified feeding or eating disorder (n = 30). No adolescents met criteria for binge eating disorder. Results: Findings showed a significant main effect of diagnosis on overall emotion dysregulation and most domains of emotion dysregulation across age groups. Adolescents with an eating disorder consistently reported higher emotion dysregulation compared to those without an eating disorder. In terms of specific behaviors, binge eating, driven exercise, and fasting were each uniquely associated with emotion dysregulation, whereas purging was not. A similar pattern was obtained within specific domains of emotion dysregulation. Findings indicate that emotion dysregulation is a key factor across eating disorder pathology, and potential treatment target among adolescents.

## A.2

### Impact of patient characteristics on clinicians’ decisions to involve dietitians in eating disorder treatment

#### Caitlin McMaster^1^, Tracey Wade^2^, Janet Franklin^3^, Glenn Waller^4^, Susan Hart^5^

##### ^1^Boden Collaboration for Obesity, Nutrition, Exercise & Eating Disorders, University of Sydney, New South Wales, Australia. ^2^Flinders University, Adelaide, South Australia, Australia. ^3^Metabolism & Obesity Services, RPA Hospital, New South Wales, Australia. ^4^University of Sheffield, Sheffield, United Kingdom. ^5^Accredited Practising Dietitian, Private Practice, New South Wales, Australia

**Correspondence**: Caitlin McMaster (caitlin.mcmaster@sydney.edu.au)

*Journal of Eating Disorders* 2021, **9(1)**: A.2

**Objective:** Dietetic involvement in eating disorder (ED) treatment is often initiated by other members of a patient’s treating team. This study aimed to examine the impact of patient characteristics on clinicians’ decisions to involve a dietitian in a patient’s treatment, as well as the influence of clinician characteristics on their decision-making.

**Method:** ED clinicians were recruited through four organisations to complete an online survey, which used case vignettes to assess their likelihood of referring patients to a dietitian or consulting with a dietitian for guidance. Measures of clinician anxiety, beliefs about the therapy they deliver, beliefs about dietetics and views on evidence-based practice where also included to determine if these were related to their responses to the case vignettes.

**Results:** Fifty-seven clinicians completed the survey, with the largest group being clinical psychologists (n = 22, 39%). ED diagnosis, weight status, medical co-morbidities and progress in treatment were all shown to be influential on whether clinicians involved dietitians. Clinician characteristics and their beliefs about dietitians were generally not correlated with the likelihood of seeking dietetic input.

**Discussion:** This study indicates that clinicians’ decisions to involve dietitians in ED treatment are systematic rather than influenced by individual characteristics of the clinician. Clinicians require further education in the potential for malnutrition regardless of patient’s ED diagnosis or weight status and the dietitian’s role in addressing this.

## A.3

### Evaluating the real-world effectiveness of a virtual group adaptation of enhanced cognitive behavioural therapy (CBT-E) in a transdiagnostic sample of youth with eating disorders

#### Sinead Day^1^, Ranjani Utpala^1^

##### ^1^Butterfly Foundation, New South Wales, Australia

**Correspondence**: Sinead Day (sinead.day@butterfly.org.au)

*Journal of Eating Disorders* 2021, **9(1)**: A.3

Developing effective virtual treatments for eating disorders has become increasingly important with the impact of the COVID-19 pandemic and growing demand for treatment services. However, no previous research has investigated the effectiveness of enhanced cognitive behaviour therapy (CBT-E) adapted for a group setting and delivered via e-health modalities. This study aimed to evaluate a 12-week, virtual outpatient group program for youth with eating disorders run by the Butterfly Foundation, an NGO providing support services to Australians struggling with disordered eating and body image concerns. Fifty-three participants aged 18–25 years (Mage = 21.6 years), with a full range of clinical and subclinical eating disorder presentations, underwent 22 biweekly sessions adapted from CBT-E. Sessions were run via Zoom and included the novel addition of a supervised snack to provide meal support in a virtual setting. The rate of treatment completion was 73.6%, which is comparable to studies of offline group CBT-E. There were significant pre- to post-group improvements in eating disorder attitudes and behaviours, as well as state emotion dysregulation, perfectionism, and self-esteem, p < .001. There was no significant change in psychological distress, p = .269. The program appeared acceptable to participants, with the majority rating it as very helpful. Overall, findings indicate that virtual group adaptations of CBT-E may be both acceptable and effective in reducing eating symptomatology and secondary outcomes for youth with diverse eating disorder presentations in real-world treatment settings. These findings have important implications for the expanding the accessibility of eating disorder treatment in order to meet rising demand for services.

## B.1

### Dietary intakes associated with addictive eating: A systematic review

#### Kirrilly Pursey^1^, Janelle Skinner^1^, Mark Leary^1^, Tracy Burrows^1^

##### ^1^University of Newcastle, New South Wales, Australia

**Correspondence**: Tracy Burrows (tracy.burrows@newcastle.edu.au)

*Journal of Eating Disorders* 2021, **9(1)**: B.1

There is rapidly growing interest in the addictive eating construct. It has been suggested that certain foods may possess addictive properties; however, the evidence for the types of foods associated with addictive eating remains unclear. This systematic review aimed to synthesise published studies assessing specific dietary intakes associated with addictive eating.

A systematic search strategy of nine databases was conducted up to November 2020 according to the PRISMA Guidelines. Studies were included if they reported associations between addictive eating, assessed via the Yale Food Addiction Scale, and dietary outcomes.

The search retrieved 5799 articles, with 16 studies included in the review. A total of 128,236 (range 18–123,688) predominantly adult (n = 13 adult studies, n = 3 child/adolescent studies) participants were included across studies. Addictive eating was associated with higher energy intake in three studies (mean difference range 540–1240 kcal), with no significant associations reported in another three studies. Addictive eating was associated with higher intakes of macronutrients including fat (n = 7 studies) and carbohydrates (n = 4 studies), and foods high in a combination of fats and refined carbohydrates (e.g., takeaway, candy); however, this was not consistent across studies.

Differences in dietary intakes were observed according to addictive eating; however, there was considerable heterogeneity due to differences in dietary assessment methods. Future treatment approaches should incorporate individualised dietary advice targeting foods high in fat and refined carbohydrates.

## B.2

### A lower carbohydrate enteral feed compared with a standard feed result in less hypophosphatemia in adolescent and young adults hospitalised with anorexia nervosa: A randomised controlled trial

#### Elizabeth Parker^1^, Victoria Flood^2^, Mark Halaki^2^, Christine Wearne^1^, Gail Anderson^1^, Linette Gomes^1^, Simon Clarke^2^, Frances Wilson^1^, Janice Russell^2^, Elizabeth Frig^3^, Michael Kohn^1^

##### ^1^Westmead Hospital, New South Wales, Australia. ^2^University of Sydney, New South Wales, Australia. ^3^Royal Prince Alfred Hospital, New South Wales, Australia

**Correspondence**: Elizabeth Parker (Elizabeth.Parker@health.nsw.gov.au)

*Journal of Eating Disorders* 2021, **9(1)**: B.2

**Aims:** Providing adequate nutrition to patients with anorexia nervosa must be balanced against concerns of developing refeeding complications, particularly the reintroduction of carbohydrate due to the risk of electrolyte, metabolic, and organ dysfunction. This study compared the efficacy and safety of an iso-caloric lower carbohydrate enteral formula (28% carbohydrate) against a standard enteral formula (54% carbohydrate).

**Methods:** Patients (aged 15–25) hospitalised with anorexia nervosa were recruited into this double blinded randomised controlled trial. At a midpoint interim 24 participants, mean age 17.5 years (± 1.1), had been randomly allocated to lower carbohydrate (n=14) or standard (n=10) feeds.

**Results:** At baseline, there was no significant difference in degree of malnutrition, medical instability, history of purging or serum phosphate levels between the two treatment arms. A significantly lower rate of hypophosphatemia developed in patients who received the lower carbohydrate formula compared to standard formula (35.7% vs. 90.0%, p=0.013). During treatment there was no significant difference in weight gain, number of days to reach medical stability, hypoglycaemia, or hospital length of stay.

**Conclusion:** The results of this randomised controlled trial suggest both feeds were equally tolerated and that weight gain was proportional to caloric prescription and not macronutrient content. Patients experienced improved metabolic health, as indicated by less hypophosphatemia, on the lower carbohydrate feed in comparison to the standard feed.

## B.3

### Exploring eating disorder-related content in accredited dietetic university programs in Australia and New Zealand

#### Elizabeth Parker^1^, Mellisa Ashley^2^, Deanne Harris^3^, Elyse Denman^4^, Courtney Moretti^4^, Anita Stefoska-Needham^4^

##### ^1^Westmead Hospital, New South Wales, Australia. ^2^Adult Eating Disorders Service, Western Sydney Local Health District, New South Wales, Australia. ^3^Hunter New England Health Local Health District, New South Wales, Australia. ^4^University of Wollongong, New South Wales, Australia

**Correspondence**: Elizabeth Parker (Elizabeth.Parker@health.nsw.gov.au)

*Journal of Eating Disorders* 2021, **9(1)**: B.3

**Background:** Dietetic students in Australia and New Zealand receive their education and training through accredited tertiary programs. Students graduate with the knowledge, skills and attributes to prepare them for practice in diverse clinical areas and settings. However, previous research indicates that dietetic students and new graduates perceive a lack of adequate training and preparedness for the treatment of individuals with eating disorders (EDs). Therefore, the present study aimed to explore the perceptions of university program convenors and lecturers towards students’ preparedness and training; specifically, towards ED content within dietetic curricula, clinical placements and other learning activities.

**Methods:** Between May 2019 and March 2021, an exploratory qualitative study, incorporating semi- structured interviews, was conducted with consenting participants. Interviews were recorded and transcribed verbatim. Four researchers independently coded and thematically analysed all transcripts. Consensus agreement was reached on dominant themes.

**Results:** Participants from 14 of 19 universities (74%), representing 19 of 24 accredited programs (79%), were involved in the study. ED-related topics were identified within all programs’ curricula, but differences existed between the depth of information and teaching approaches. A lack of clinical placement opportunities was a common theme.

**Conclusion:** Accredited dietetics programs do incorporate ED content into their curricula, primarily focusing on nutrition assessment skills. Further training after graduation is recommended to provide safe, effective and evidence-informed treatment to individuals with Eds.

## B.4

### Exploring perceived training and professional development needs of Australian dietetics students and practising dietitians in the area of eating disorders: A focus group study

#### Courtney Moretti^1^, Elizabeth Parker^2^, Mellisa Ashley^3,2,^ Deanne Harris^4^, Anita Stefoska-Needham^1^

##### ^1^University of Wollongong, New South Wales, Australia. ^2^Westmead Hospital, New South Wales, Australia. ^3^Adult Eating Disorders Service, Western Sydney Local Health District, New South Wales, Australia. ^4^Hunter New England Health Local Health District, New South Wales, Australia

**Correspondence**: Elizabeth Parker (Elizabeth.Parker@health.nsw.gov.au)

*Journal of Eating Disorders* 2021, **9(1)**: B.4

**Background:** Training of health professionals has been shown to effectively increase their confidence to practice with patients with an eating disorder (ED). However, the ED training needs of Australian dietitians are yet to be determined. The aim of this study was to explore the perceptions of practising dietitians and dietetic students towards training in the area of Eds to identify any gaps in their current training and opportunities for future professional development.

**Methods:** A qualitative descriptive study was conducted, incorporating six virtual focus groups (FGs) involving practising dietitians and dietetic students in Australia. FGs were recorded, transcribed verbatim, coded and then thematically analysed independently by 4 researchers. Consensus agreement was reached on the dominant themes.

**Results:** Thirty-eight participants (26 dietitians, 12 students) were recruited, mean age 32.5 ± 11 years. Three major themes emerged: (1) dietitians’ perceptions of under-preparedness impact their confidence to work with patients with an ED; (2) both personal and organisational factors are challenges to dietetic practice with this patient group; and (3) further training and professional supervision is at the centre of the solution.

**Conclusions:** Dietitians and student dietitians identified that further training is an integral solution to improving both confidence and mental health literacy when working with patients with an ED. Further research is recommended to clarify the specific type of training required by dietitian.

## C.1

### Establishing current eating disorder research and translation priorities for Australia

#### Peta Marks^1^, Danielle Maloney^1^, Ashlea Hambleton^1^, Phillip Aouad^1^, Shannon Calvert^2^, Belinda Caldwell^3^, Beth Shelton^4^, Siân McLean^5^, Stephen Touyz^1^, Sarah Maguire^1^.

##### ^1^InsideOut Institute, Sydney University, New South Wales, Australia. ^2^Healing Conversations, Perth, Western Australia, Australia. ^3^Eating Disorders Victoria, Northcote, Victoria, Australia. ^4^NEDC, Brunswick, Victoria, Australia. ^5^La Trobe University, Melbourne, Victoria, Australia

**Correspondence**: Peta Marks (peta.marks@sydney.edu.au)

*Journal of Eating Disorders* 2021, **9(1)**: C.1

In 2020, InsideOut Institute undertook a study to capture the current research and translation priorities for Australia, in association with the UK’s James Lind Alliance (JLA) whose consultant oversaw the process (with ethics approval from Sydney LHD). The JLA method is internationally recognised and responds to the health care and research paradigm shift towards focusing on the needs of people impacted by particular conditions. JLA aims to identify and prioritise unanswered research priorities with those affected, caregivers and practitioners. In this project, national consultations collected 1210 plain language questions, which were categorised, merged, summarised and checked against existing evidence. A national survey including 431 participants (160 consumers, 140 clinicians, 52 researchers, 79 carers), established an interim priority list. The top 16 prioritised questions were taken to a consensus workshop process, facilitated by the JLA, including equal representation of consumers, carers, clinicians and researchers, with advisory committee observers. The current ‘Top 10’ questions—focusing on symptom identification, treatment access and outcomes, risk factors, and workforce capability, were finalised and now require communication, discussion and debate—with government, community, researchers and potential funders—to ensure issues that matter most to frontline clinicians, consumers and carers are raised for exploration, and ultimately help inform the research agenda.

## C.2

### Critical eating disorders research: Looking to the past and creating new paths forward

#### Andrea LaMarre^1^

##### ^1^Massey University, Auckland, New Zealand

**Correspondence**: Andrea LaMarre (a.lamarre@massey.ac.nz)

*Journal of Eating Disorders* 2021, **9(1)**: C.2

What does it mean to do “critical” eating disorders research? Does it mean tossing the baby out with the bathwater, doing away with all mention of randomised controlled trials or statistical significance? Does it mean denying eating disorders as a clinical entity? Does it mean complaining all the time? In this presentation, I will explore the meanings of “critical eating disorders research,” drawing on examples from pathbreaking critical feminist scholars whose work does not always get heard in eating disorders journals and conferences. I will offer practical guidance around how this approach to eating disorders research allows us to open to different ways of being, inviting not simply complaint but ways to generate findings that make a difference in people’s lives and relationships with food and body. Highlighting emerging arts-based work on eating disorders, I will illustrate the importance of theoretical and methodological alignment in social justice oriented critical eating disorders work in particular. A key contention at the heart of this presentation is that if we wish to know something new about eating disorders, we need to use different methods and invite and centre difference in our research studies.

## C.3

### Investigating an interpersonal model of eating disorder symptoms

#### Katelin Bullman^1^, Elizabeth Rieger^1^, Richard Burns^1^

##### ^1^Australian National University, Australian Capital Territory, Australia

**Correspondence**: Katelin Bullman (katelin.bullman@anu.edu.au)

*Journal of Eating Disorders* 2021, **9(1)**: C.3

**Objective:** There currently exists a research gap regarding which interpersonal factors should be targeted to improve treatment outcomes for individuals with an eating disorder. To address this, an eating disorder-specific model of interpersonal psychotherapy (IPT-ED) was designed to identify the relevant interpersonal factors that drive eating disorder symptoms and hence should be targeted in treatment. The present study sought to provide a comprehensive evaluation of the validity of the IPT-ED model as well as testing possible refinements of the model.

**Method:** Females between the age of 18–30 years (N = 321) completed online self-report questionnaires examining the key constructs of the IPT-ED model, including negative social evaluation (both general and specific to body shape/weight), low self-esteem, negative affect, outcome expectancies regarding eating, and eating disorder symptoms.

**Results:** Structural equation modelling indicated that the relationship between negative social evaluation and eating disorder symptoms was fully mediated by low self-esteem and negative affect. In addition, the relationship between negative affect with eating disorder symptoms was moderated by outcome expectancies regarding eating. Finally, eating disorder symptoms in turn predicted negative social evaluation.

**Conclusion:** Results were generally supportive of the validity of the IPT-ED model, but also suggested that some refinements may be needed to more accurately inform interventions aimed at preventing and treating eating disorders.

## C.4

### What is OSFED? The predicament of classifying residual eating disorders

#### Zoe Jenkins^1,2,3^, Andrea Phillipou^1,2,3^, David Castle^2,3^

##### ^1^Swinburne University of Technology, Victoria, Australia. ^2^University of Melbourne, Victoria, Australia. ^3^St Vincent's Hospital, Victoria, Australia

**Correspondence**: Zoe Jenkins (Zoe.JENKINS@svha.org.au)

*Journal of Eating Disorders* 2021, **9(1)**: C.4

**Objective:** The DSM-5 relaxed diagnostic criteria for anorexia nervosa (AN) and bulimia nervosa (BN), and recognised a third eating disorder (ED); binge eating disorder (BED). However, a large proportion of cases remain in the residual category of ‘other specified feeding and eating disorders’ (OSFED), which contains substantial heterogeneity. We aimed to investigate whether the OSFED could be classified further by subdividing based on dominant clinical feature; purge or restraint.

**Methods:** Data from 390 patients attending an outpatient ED treatment service were analysed. Diagnostic algorithms were derived from DSM-5 criteria using scores on items from the Eating Disorder Examination-Questionnaire (EDE-Q) to classify individuals into diagnostic groups: AN, BN, BED and OSFED. Cluster analyses were performed on the OSFED group, based on BMI and symptoms of restraint, binge eating, purging and over-evaluation of shape and weight.

**Results:** 232 (59.5%) patients had a diagnosis of AN, 87 (22.3%) BN, 13 (3.3%) BED and 132 (33.8%) OSFED. Cluster analysis failed to identify clusters that represented the OSFED group as a whole. An overview of the clinical characteristics (BMI, EDE-Q subscales) of the OSFED patients will be summarised.

**Discussion:** The current study failed to identify specific symptom clusters that represented the OSFED group as a whole. However, it highlighted that some individuals do not endorse the ED behaviours currently assessed by the DSM-5.

## D.1

### Positive outcomes from integrating telehealth into routine clinical practice for eating disorders during COVID-19

#### Bronwyn Raykos^1^, David Erceg-Hurn^1^, James Hill^1^, Bruce Campbell^1^, Peter McEvoy^1,2^

##### ^1^Centre for Clinical Interventions, Western Australia, Australia. ^2^Curtin University, Western Australia, Australia

**Correspondence**: Bronwyn Raykos (bronwyn.raykos@health.wa.gov.au)

*Journal of Eating Disorders* 2021, **9(1)**: D.1

**Background:** The coronavirus pandemic (COVID-19) has required telehealth to be integrated into the delivery of evidence-based treatments for eating disorders in many services, but the impact of this on patient outcomes is unknown.

**Objective:** The present study examined the impact of the first wave of COVID-19 and rapid transition to telehealth on eating disorder symptoms in a routine clinical setting.

**Method:** Participants were 25 patients with a confirmed eating disorder diagnosis who had commenced face-to-face treatment and rapidly switched to telehealth during the first wave of COVID-19 in Western Australia, Eating disorder symptoms, clinical impairment and mood were measured prospectively before and during COVID-19. Patients also completed a retrospective survey to understand their perceptions of telehealth and COVID-19.

**Hypotheses:** We predicted that: (1) patients would experience worsening eating disorder symptoms during COVID-19 and (2) patients would perceive poorer quality of treatment and worsening eating disorder symptoms during COVID-19 and switch to telehealth.

**Results:** Neither hypothesis was supported. On average, patients achieved large improvements in eating disorder symptoms and mood, and the magnitude of improvement in eating disorder symptoms was comparable to historical benchmarks at the same clinic.

**Discussion:** Providing evidence-based treatment for eating disorders via telehealth during COVID-19 lockdown.

## D.2

### The impact of the COVID-19 pandemic on people with lived experience of an eating disorder: An Australian perspective

#### Jane Miskovic-Wheatley^1^, Eyza Koreshe^1^, Marcellinus Kim^2^, Rachel Simeone^1^, Sarah Maguire^1^

##### ^1^InsideOut Institute, University of Sydney, New South Wales, Australia. ^2^Sydney Local Health District, New South Wales, Australia

**Correspondence**: Jane Miskovic-Wheatley (Jane.miskovic-wheatley@sydney.edu.au)

*Journal of Eating Disorders* 2021, **9(1)**: D.2

**Background:** Associated with the COVID-19 pandemic is an emerging mental health crisis. People with a lived experience of eating disorders (ED) may be particularly vulnerable due to exasperating impact of the virus, co-occurring conditions, and necessary public health measures. This study investigates the association of the pandemic with ED symptoms to identify risk factors for clinical consideration.

**Method:** A national online observational survey was conducted due to reach, cost-effectiveness, safety, and suitability in Australia from May to October 2020 to 6 month follow up.

**Results:** 1723 participants completed the survey, mode age 24.93 years (16–80 yrs), majority female (91.58%). Nearly 90% reported increased body image concern, 74.75% increased food restriction, and 72.10% increased binging. Higher Eating Disorder Examination Questionnaire Global Scores were associated with increased dieting, binging, purging, over-exercise and laxative misuse, with higher state depression, anxiety, stress and loneliness associated with increase in a variety of ED concerns and behaviours. Generalised public health messaging was associated with increased ED symptomatology, and an increased need for supports was determined.

**Conclusion:** This study highlights the need for increased clinical awareness of the risk factors and targeted intervention for people in the community with a lived experience of ED, including those not formally diagnosed or currently engaged in targeted treatment services, during this global public health crisis.

## D.3

### Eating disorder treatment in the time of COVID: The experience of telehealth

#### Jane Miskovic-Wheatley^1^, Eyza Koreshe^1^, Rachel Simeone^1^, Sarah Maguire^2^

##### ^1^InsideOut Institute, University of Sydney, New South Wales, Australia

**Correspondence**: Jane Miskovic-Wheatley (Jane.miskovic-wheatley@sydney.edu.au)

*Journal of Eating Disorders* 2021, **9(1)**: D.3

**Background:** The COVID-19 pandemic changed provision of health care such as disruption to established treatment and widespread use of telehealth services. Evidence suggests efficacy of telehealth for CBT and FBT, however with 51 M telehealth services delivered to 13 M patients across health care by March 2021, the safety, efficacy and experience of telehealth for a higher-risk population with a lived experience of eating disorders (ED) must be evaluated.

**Method:** A national online observational survey (quantitative and qualitative) was conducted May- October 2020 for people with ED to record symptom and treatment experience.

**Results:** Of 1723 respondents, 468 were in regular treatment for ED: 48% transferred to telehealth, 17% experienced treatment disruption, and 6% were unable to receive regular treatment. 1540 respondents had at least one telehealth service: 62% preferred in-person treatment, 30% a mixture of treatment modalities, 3% preferred telehealth. Of the cohort, 38% did not have personal infrastructure required for telehealth services, and for those engaged in treatment, over half reported they did not understand how their safety would be monitored by providers.

**Conclusion:** There has been call for more accessible options for people with ED, such as telehealth for rural communities, a need accelerated into provision during the pandemic. Ongoing evaluation of safe and effective practice, considering accessibility and patient experience is critical, with insights provided.

## D.4

### Enhancing help seeking with support from a telehealth nurse

#### Belinda Caldwell^1^

##### ^1^Eating Disorders Victoria, Victoria, Australia

**Correspondence**: Belinda Caldwell (belinda.caldwell@eatingdisorders.org.au)

*Journal of Eating Disorders* 2021, **9(1)**: D.4

In 2019, Eating Disorders Victoria employed a Telehealth Nurse to provide Victorians with an eating disorder (and/or their carers) support with finding a clinical team, connecting members of the team together and supporting clients to engage in help-seeking through written actions plans. In July 2020 EDV undertook an evaluation the first year of the program.

**Objective:** The objective of the evaluation was to assess if EDV’s Telehealth Nurse Program is achieving its aims in terms of persons reached and increasing the capacity of people with eating disorders and their support people, to navigate the health system.

**Methodology:** Client service statistics were retrieved from the EDV databases and a survey was undertaken with 156 EDV Telehealth Nurse contacts.

**Findings:** EDV connected with 208 new referrals and had 962 contacts in 12 months. Survey data showed EDV provided 77% of participants with a written action plan, of which 71% reported seeing improvements and 82% of participants said they know how and felt confident to move forward in their/their loved one’s recovery.

The findings showed that the Telehealth Nurse program has increased the capacity of people with eating disorders and their carers, to seek help. Participants have had learning opportunities and skills development, been linked to health system networks, received resources and developed pathways to work towards their/their loved one’s recovery.

## D.5

### The EDV Hub: The first step for recovery

#### Rebecca Lister^1^

##### ^1^Eating Disorders Victoria, Victoria, Australia

**Correspondence**: Rebecca Lister (rebecca.lister@eatingdisorders.org.au)

*Journal of Eating Disorders* 2021, **9(1)**: D.5

Eating Disorders Victoria’s (EDV) Hub is a free and confidential service that provides information, navigation and support for people experiencing eating disorders or those who are supporting them. Using a peer support model, trained volunteers offer a safe space to connect, openly discuss eating disorders and it is often the first step in a person’s recovery.

During the COVID-19 pandemic, enquiries to the Hub increased twofold. Volunteers and staff were operating the service from their homes and a new record management system was introduced to capture the contacts and track their recovery. EDV has developed an evaluation framework and reporting system for the Hub data based on social impact methodology. A theory of change has captured short, medium and long-term change.

This presentation will go into detail about the service, the impact of COVID-19 and current evaluation results.

## D.6

### The ‘zoom effect’: Exploring the impact of video-calling on appearance dissatisfaction during the COVID-19 pandemic

#### Toni Pikoos^1^, Simone Buzwell^1^, Gemma Sharp^2^, Susan Rossell^1^

##### ^1^Swinburne University of Technology, Victoria, Australia. ^2^Monash University, Victoria, Australia

**Correspondence**: Toni Pikoos (tonipikoos@gmail.com)

*Journal of Eating Disorders* 2021, **9(1)**: D.6

**Background:** The popularity of videoconferencing platforms has skyrocketed during the COVID-19 pandemic, however, there have been concerns regarding the potential for video calls to promote appearance dissatisfaction, as individuals are exposed to their reflection on camera for extended periods. The current study characterised current video-usage behaviours and their relationship with appearance dissatisfaction and interest in beauty procedures in the general population.

**Method:** An online survey was completed by 379 adults living in Australia in May or July 2020 during COVID-19 lockdown. Multiple aspects of video usage were assessed, including engagement in ‘video- manipulation’ techniques to enhance appearance and the focus of visual attention while on video calls (i.e., on self or others). The Dysmorphic Concern Questionnaire was administered to determine if video-use behaviours were associated with greater body image disturbance.

**Results:** Over one-third of participants had identified new appearance concerns while on video. Dysmorphic concern was associated with self-focused attention, video manipulation behaviours, and increasing appearance concerns. Individuals who identified new video-based appearance concerns reported greater interest in obtaining future beauty and cosmetic procedures.

**Discussion:** This is one of first studies to report the potential risks of video call usage on body image and appearance dissatisfaction. Further research is needed to understand how best to mitigate these risks, as COVID-19 accelerates a virtual age of communication.

## E.1

### Predictors of domains of recovery in adolescents with eating disorders

#### Emily Jones^1^

##### ^1^Curtin University, Western Australia, Australia

**Correspondence**: Emily Jones (Emily.j.jones@postgrad.curtin.edu.au)

*Journal of Eating Disorders* 2021, **9(1)**: E.1

Eating disorders can be severe chronic illnesses; therefore, identifying factors that predict domains of recovery is important. The present study investigated the transdiagnostic cognitive-behavioural model of eating disorders to investigate predictors of cognitive, behavioural, and physical dimensions of recovery in adolescents. Using a longitudinal design, adolescents with eating disorders (N = 172 [94.8% female, M = 14.79 years, SD = .80]) completed measures of perfectionism, self-esteem, mood intolerance, interpersonal difficulties, and eating disorder symptoms as part of an intake assessment and 6-month clinical review assessment at an eating disorder program. Perfectionism, self-esteem, mood intolerance, and interpersonal difficulties significantly predicted eating disorder symptoms and binge eating frequency. Self-esteem, mood intolerance, and interpersonal difficulties significantly predicted frequency of purging behaviour. Perfectionism was the only significant predictor of body mass index, however not in the hypothesised direction. The findings suggest support for the transdiagnostic model of eating disorders and continued examination of cognitive-behaviour therapy-enhanced (CBT-E) to aid recovery from eating disorders.

## E.2

### Neurodivergence and eating disorders: Zooming in on sensory processing

#### Laurence Cobbaert^1^, Ruth Minkov^2^, Meghan Zadow^3^

##### ^1^University of New South Wales, New South Wales, Australia. ^2^University of Melbourne, Victoria, Australia. ^3^University of Adelaide, South Australia, Australia

**Correspondence**: Laurence Cobbaert (l.cobbaert@unsw.edu.au)

*Journal of Eating Disorders* 2021, **9(1)**: E.2

Recent research is evidencing strong links between autism, ADHD, and eating disorders. Autism and ADHD both involve atypical sensory processing and are under-recognised in females. Atypical sensory processing is shown to affect body image and emotional liability. In other words, sensory sensitivity is inherently interwoven with self-perception and self-regulation. The overarching role of sensory processing is further highlighted in other cognitive processes such as executive functioning, anxiety, impulsivity, compulsiveness, and obsessiveness. The notion that a lot of individuals with eating disorders tend to be perfectionistic high achievers while also often having a variety of artistic talents is well-established. Creativity and high achieving potential both strongly correlate with heightened exteroception—also referred to as overexcitabilities. Based on those findings, we argue that sensory processing is an important mediator underlying co-occurrence between eating disorders and abovementioned neurodevelopmental phenomena. Our innovative perspective has implications for clinical practice as current therapy frameworks anchored in CBT fail to acknowledge the influence of sensory sensitivity on self-perception, behaviours, and cognition. Therefore, we believe that therapy should be informed by sensory profiles to simultaneously address sensory needs, emotional responsiveness, and executive functioning. This perspective also has implications for prevention efforts: assessing children’s sensory profile as part of school curriculum might provide a way of predicting those at the highest risk of developing clinically significant eating disorders.

## F.1

### Central coherence and set-shifting between non-underweight eating disorders and anorexia nervosa: A systematic review and meta-analysis

#### Ella Keegan^1^, Kate Tchanturia^2,3^, Tracey Wade^1^

##### ^1^Flinders University, South Australia, Australia. ^2^Department of Psychological Medicine, King’s College, United Kingdom. ^3^Eating Disorder Service South London and Maudsley NHS Trust, United Kingdom

**Correspondence**: Ella Keegan (ella.keegan@flinders.edu.au)

*Journal of Eating Disorders* 2021, **9(1)**: F.1

**Background:** Central coherence and set-shifting inefficiencies have been identified as possible maintaining factors in anorexia nervosa (AN). However, little attention has been paid to central coherence and set-shifting in non-underweight eating disorders, that is, bulimia nervosa (BN) and binge eating disorder (BED). If this group has inefficiencies comparable to those in AN then they might benefit from adjunct cognitive remediation therapy (CRT).

**Aims:** We examined whether people with non-underweight eating disorders have central coherence and set-shifting inefficiencies, and tested whether their performance differs from people with AN.

**Method:** We performed random effects meta-analyses on 16 studies (1112 participants) for central coherence and 38 studies (3,505 participants) for set-shifting.

**Results:** We found evidence of central coherence inefficiencies in AN (g = − 0.53, 95% CI: − 0.80, − 0.27, p < .001) and BN (g = − 0.70, 95% CI: − 1.14, − 0.25, p = .002) but not BED. Similarly, we found evidence of set-shifting inefficiencies in AN (g = − 0.38, 95% CI: − 0.50, − 0.26, p < .001) and BN (g = − 0.55, 95% CI: − 0.81, − 0.29, p < .001) but not BED. The effect sizes for non-underweight eating disorders did not differ from those for AN (ps > .05).

**Conclusions:** We were underpowered to make definitive conclusions about BED. However, central coherence and set-shifting inefficiencies are clearly present in BN and do not differ from those in AN. Clinically, this suggests that people with BN might benefit from adjunct CRT.

## F.2

### Anorexia nervosa and unaffected sisters: Similarities and differences

#### Andrea Phillipou^1^, Caroline Gurvich^2^, David Castle^3,4^, Susan Rossell^1^

##### ^1^Swinburne University of Technology, Victoria, Australia. ^2^Monash Alfred Psychiatry Research Centre, Alfred Hospital, Victoria, Australia. ^3^University of Melbourne, Victoria, Australia. ^4^St Vincent's Hospital, Victoria, Australia

**Correspondence**: Andrea Phillipou (andreaphillipou@swin.edu.au)

*Journal of Eating Disorders* 2021, **9(1)**: F.2

Research has indicated the unaffected siblings of individuals with anorexia nervosa (AN) often display similarities in psychological symptoms and cognitive performance, suggesting that these characteristics may be familial. The aim of this study was to examine a range of psychological characteristics (eating disorder symptoms, negative mood and perfectionism) and cognitive performance (cognitive flexibility) between individuals with a current diagnosis of AN (c-AN), individuals’ weight-restored from AN (wr-AN), sisters of individuals with AN (AN-sis) and healthy controls (HC). Eighty female participants (n = 20/group; age M = 23.31, SD = 3.92) were recruited and tested at Swinburne University. The results indicated that eating disorder symptoms as well as depression and stress were significantly higher for the c-AN group than AN-sis, but were similar between AN-sis and wr-AN groups (p < .001). c-AN, wr-AN and AN-sis groups did not significantly differ to one another on anxiety, but all scored higher than HC (p < .001). The two AN groups reported increased perfectionism to AN-sis and HC who had similar results (p < .001). Cognitive flexibility was similar across all groups (Bonferroni-corrected, p > .01). The findings indicate that a number of similarities exist between individuals with AN and unaffected siblings, suggesting a potential familial link indicative of trait characteristics.

## F.3

### A life within stillness: Illuminating severe enduring anorexia nervosa

#### Laura Kiely^1^, Phillipa Hay^1^, Janet Conti^1^

##### ^1^Western Sydney University, New South Wales, Australia

**Correspondence**: Laura Kiely (laura.kiely@iinet.net.au)

*Journal of Eating Disorders* 2021, **9(1)**: F.3

It is said that a picture paints a thousand words, and we are indeed in need of words to better understand SE-AN. One in five people with AN develop an enduring course of illness, presenting the highest mortality rate of any mental illness with significant social/personal impact. This has prompted an urgent call to action to better understand, classify and treat SE-AN and prevent a protracted course of illness (Wonderlich 2020).

Through a single case report with interpretive phenomenological thematic analysis, this research aims to illuminate aspects of a lived experience with SE-AN and identity negotiations, as expressed through recorded dialogue and visual, artistic representation.

The artist’s works, considered alongside their verbalised meanings, display inherent polarities and contradictions, present with shame, distortion and fragmentation, juxtaposed with an undeniable beauty, grace and wisdom. A selection of the preliminary findings in this qualitative research will be considered in the context of other author’s findings to build upon the emerging picture of SE-AN and seek to contribute to understanding people’s experiences and the ways they construct a sense of identity with long-standing anorexia nervosa. More broadly, to canvas novel ideas and stimulate discussion around nosology, identification and ethical considerations in the treatment of AN and its enduring form.

## F.4

### The language of recovery from eating disorders: Thematic analysis of ‘your own words’

#### Elysa Roberts^1^, Chloe Weaver^1^, Anna Rose^1^

##### ^1^University of Newcastle, New South Wales, Australia

**Correspondence**: Elysa Roberts (elysa.roberts@newcastle.edu.au)

*Journal of Eating Disorders* 2021, **9(1)**: F.4

The language used by individuals to describe recovery from eating disorders can be characterised by imagery, metaphor and experiences of setback and progress. Such qualitative accounts from lived experiences can be informative. Our study explored the language of recovery in responses to the prompt, ‘describe your stage of recovery in your own words’ via online survey. Responses were from 361 adults in Australia, Canada, the USA and the UK at various stages of recovery from anorexia nervosa, bulimia nervosa and binge eating disorder. Data were analysed using thematic analysis. Findings reflect a tabulation of patterns of language and consolidation of themes and subthemes. One member of the research team has a lived experience of recovery and another had close exposure to the recovery experience. A third researcher had little direct involvement with eating disorder experiences, which offered a form of internal audit. Findings revealed an overarching theme, The Language of Recovery, with four subthemes. The preponderance of conjunctive phrases, e.g., ‘but’ or ‘but still’, used in one subtheme to qualify a negative or positive state of recovery was provocative. Another subtheme reflected ways respondents used language to describe recovery evocatively and figuratively. Additional subthemes denoted how respondents qualified or quantified their stage of recovery. Implications of results are discussed in the context of current literature, clinical practice, and the recovery experience.

## G.1

### Beach body ready? Shredding for summer? A first look at seasonal body image and seasonal dieting around the world

#### Scott Griffiths^1^, Emma Austen^1^, Isabel Krug^1^, Khandis Blake^1^

##### ^1^University of Melbourne, Victoria, Australia

**Correspondence**: Scott Griffiths (scott.griffiths@unimelb.edu.au)

*Journal of Eating Disorders* 2021, **9(1)**: G.1

We introduce the terms seasonal body image and seasonal dieting to refer to within-person variation in body image and dieting that occur across Spring, Summer, Autumn, and Winter. In our first study, we visualised seasonal body image and its mechanisms by analysing cross-sectional data from sexual minority men (N = 823 in 4 countries) who answered questions about their experiences of body image phenomena in each season. As hypothesised, in Summer we observed peaks for body dissatisfaction alongside peaks in four seasonal body image mechanisms: pressure from media advertisements, pressure from peers on social media, the feeling that one’s body is on public display, and appearance comparisons. In Winter, these phenomena were weakest/lowest. In our second study, we visualised seasonal dieting by analysing longitudinal changes in the frequency of dieting-related tweets in a geo-located Twitter database (N ≈ 6 billion) spanning six calendar years and ≈190 countries. A seasonal effect emerged: as the weather got hotter from Winter to Spring, and from Spring to Summer, the frequency of dieting tweets increased. Effect sizes across both studies ranged from small to large (rs = .07–.50) with an average effect size of medium (.38). Taken together, our findings suggest that seasonal social and cultural pressures have substantive impacts on the body image and dieting behaviour of individuals in countries worldwide.

## G.2

### Prevalence of body dissatisfaction in early adolescent boys and girls: Association with depressive symptoms and body change strategies

#### Siân McLean^1^, Rachel Rogers^2^, Amy Slater^3^, Hannah Jarman^1^, Chloe Gordon^4^, Susan Paxton^1^

##### ^1^La Trobe University, Victoria, Australia. ^2^North-eastern University, Boston, USA. ^3^The University of the West of England, Bristol, UK. ^4^Australian Catholic University, Victoria, Australia

**Correspondence**: Siân McLean (s.mclean@latrobe.edu.au)

*Journal of Eating Disorders* 2021, **9(1)**: G.2

Body dissatisfaction is a public health concern and risk factor for eating disorders. Although adolescence is a high-risk period for onset of body dissatisfaction, data on prevalence and severity of body dissatisfaction are lacking, particularly for boys. This study aimed to describe the prevalence in adolescents of low, moderate, and clinically significant body dissatisfaction and associations with depressive symptoms and body change strategies. Boys (n = 367; Mage = 12.8, SD = 0.7) and girls (n = 368; Mage = 12.7, SD = 0.7) completed assessments of body dissatisfaction, depressive symptoms, dietary restraint, and engagement in strategies to increase muscularity. Significantly higher proportions of girls (19.8%) than boys (6.8%) had clinically significant body dissatisfaction, whereas boys (37.9%) were more likely to have moderate body dissatisfaction than girls (20.7%). There was no gender difference in no/low body dissatisfaction (boys 55.3%; girls 57.6%). Older adolescents were more likely than younger adolescents to have clinically significant body dissatisfaction. Adolescents with clinically significant body dissatisfaction were 24 times more likely to also have possible-, probable-, or major-depressive episodes than adolescents with no/low body dissatisfaction and also had significantly higher dietary restraint and engagement in strategies to increase muscle size. Findings indicate that adolescents with clinically significant body dissatisfaction are especially at risk for experiencing depressive symptoms. Identification of and early intervention for body dissatisfaction are crucial to reduce the burden on adolescents, especially girls.

## G.3

### Disordered eating, body image concerns and weight control behaviours in children: A systematic review and meta-analysis of universal-selective prevention interventions

#### Kirrilly Pursey^1^, Tracy Burrows^1^, Daniel Barker^1^, Melissa Hart^2^, Susan Paxton^3^

##### ^1^University of Newcastle, New South Wales, Australia. ^2^Hunter New England Health, New South Wales. ^3^La Trobe University, Victoria, Australia

**Correspondence**: Tracy Burrows (tracy.burrows@newcastle.edu.au)

*Journal of Eating Disorders* 2021, **9(1)**: G.3

Body image concerns and extreme weight control behaviours frequently develop in childhood indicating that this may be an important age for the implementation of prevention approaches. This systematic review aimed to evaluate the effect of universal-selective prevention interventions addressing disordered eating, body image concerns and/or extreme weight control behaviours in children aged 6–12 years. A systematic search strategy of nine databases was conducted up to April 2020 in line with the PRIMSA guidelines. Studies were included if they delivered a universal-selective prevention intervention to children aged 6–12 years and reported outcomes relating to body image, disordered eating, or weight control behaviours. The search strategy retrieved 12,146 studies, with 38 studies included in the review. Most studies (n = 24; 60%) were classified as neutral quality and there was significant variability across interventions included in the review. Meta-analysis (n = 15 studies) revealed a trend towards improvement in body image-related outcomes across all studies that approached statistical significance (SMD 0.26 [95%CI − 0.01, 0.53]) with a high level of heterogeneity (I^2 = 91.1%; p < 0.01). Meta-analysis according to gender revealed a general improvement in body image-related outcomes for girls (SMD 0.40 [95%CI 0.07, 0.73]), but not boys (SMD 0.23 [95%CI − 0.24, 0.70]). There was a trend towards improvements in body image-related variables, particularly in girls. Future directions include the use of high-quality study designs and the inclusion of more diverse samples.

## G.4

### Body image profiles according to body shame, body appreciation and body mass index (BMI), differentiate dietary restraint and engagement amount

#### Anita Raspovic^1^, Ivanka Prichard^2^, Zali Yager^3^, Laura Hart^4^

##### ^1^La Trobe University, Melbourne, Australia. ^2^Flinders University, South Australia, Australia. ^3^Victoria University, New South Wales, Australia. ^4^University of Melbourne, Victoria, Australia

**Correspondence**: Anita Raspovic (a.raspovic@latrobe.edu.au)

*Journal of Eating Disorders* 2021, **9(1)**: G.4

Body image motivates eating and exercise patterns, however a lack of research investigating specific relationships and the influence of body morphology, limit our ability to enhance health behaviours through improvements to body image. Using latent profile analysis (LPA), this study identified body image profiles (BIPs) according to relative proportions of body shame, body appreciation and body mass index (BMI) and investigated whether these profiles differentiated dietary restraint and weekly exercise amount among adult women (N = 1200; Mage = 40.96, SD +/− 10.70). LPA revealed a four-class model as most parsimonious. The 4 BIPs were: an Appreciative-BIP which had the lowest BMI, a Medium Shame-BIP with the second lowest BMI, a High Shame-BIP which had the highest BMI and an Average-BIP with the second highest BMI. Dietary restraint and exercise amount were significantly different according to BIP in all but two comparisons. Women in the High Shame-BIP reported the highest dietary restraint and lowest weekly exercise, which was the inverse for the Appreciative-BIP. Study findings support the notion that body image is a complex co-valenced construct and extend them by showing how BMI intersects with body image to form unique profiles. Importantly, BIPs differentiated adaptive versus potentially maladaptive dietary restraint practices and exercise amounts. This research has important public health implications for the holistic promotion of healthful diet and exercise.

## G.5

### Body dissatisfaction, beauty ideals and perceived attainability of those ideals in adolescent girls: A cross-cultural study

#### Vani Kakar^1^, Jasmine Fardouly^2^, Ron Rapee^1^, Soroor Arman^3^, Mingchun Guo^4^, Elham Niazi^3^

##### ^1^Macquarie University, New South Wales, Australia. ^2^University of New South Wales, New South Wales, Australia. ^3^Isfahan University of Medical Sciences, Isfahan, Iran. ^4^Fujian Normal University, Shangjie Town, China

**Correspondence**: Vani Kakar (vani.kakar@mq.edu.au)

*Journal of Eating Disorders* 2021, **9(1)**: G.5

Research suggests that unattainable female beauty ideals can increase body dissatisfaction among girls. However, little research has investigated how body image concerns, body ideals, and attainability of those ideals, fluctuate cross-culturally. This study surveyed 790 adolescent girls living in Australia (n = 184), China (n = 294), India (n = 222), and Iran (n = 200), assessing their body satisfaction, body ideals, and perceived attainability of those ideals. Adolescent girls in Australia were the least satisfied with their bodies, followed by girls in China, and then girls in India and Iran. Discrepancy between perception of their own body image and perception of the ideal body image was highest for girls living in Iran, followed by girls in China, Australia and was most congruent for Indian adolescents. Perceived attainability of the ideal was highest for Indian and Iranian adolescents followed by girls living in Australia, and then China. Perceived attainability was positively associated with body satisfaction and was strongest for Australian adolescents followed by girls from India, China, and Iran. Perceived attainability was negatively associated with discrepancy in body image for all countries except Iran. Culture is an important factor to investigate when understanding the development of body dissatisfaction, beauty ideals, and perceived attainability of those ideals.

## H.1

### Pilot evaluation of an eating disorders quality improvement tool for Primary Health Networks in Australia

#### Louise Dougherty^1^, Beth Shelton^1^

##### ^1^National Eating Disorders Collaboration, Victoria, Australia

**Correspondence**: Louise Dougherty (louise.dougherty@nedc.com.au)

*Journal of Eating Disorders* 2021, **9(1)**: H.1

**Background:** Operating as service commissioners and system integrators, the federally funded Primary Health Networks (PHNs) in Australia have an important role in enhancing early intervention and improving access to high-quality primary care services for people with eating disorders. The National Eating Disorders Collaboration (NEDC) has developed an interactive Eating Disorders Quality Improvement (ED-QI) tool, to support PHNs to improve their eating disorders response.

**Aims:** A pilot study was conducted to identify (a) if the tool assisted PHNs to identify, prioritise and plan service improvements and (b) enablers and barriers to implementation.

**Methods:** Seven PHNs participated in the pilot over an eight-week period. A mixed-methods approach was utilised. Semi-structured interviews were conducted at three time points and participants completed an online evaluation survey at the end of the pilot. Quantitative data was analysed using descriptive statistics and qualitative data was analysed through content analysis.

**Results:** On average, participants gave the tool a rating of 8/10 for assisting in identifying, prioritising and planning service improvements. It was considered well-matched to the role of PHNs. Implementation enablers included provision of NEDC resources and external support. Barriers included lack of capacity and difficulty engaging others in change.

**Conclusion:** The ED QI tool is a useful resource for equipping PHNs to address eating disorders in their region. Findings will support roll-out of the tool across Australia.

## H.2

### Prevalence of disordered eating in a cohort of young Australians presenting for treatment at a community youth mental health service

#### Amy Burton^1^, Blake Hamilton^1^

##### ^1^University of Sydney, New South Wales, Australia

**Correspondence**: Amy Burton (amy.burton@sydney.edu.au)

*Journal of Eating Disorders* 2021, **9(1)**: H.2

headspace is the National Youth Mental Health Foundation in Australia. headspace centres aim to offer early intervention for mild to moderate mental health disorders for young people aged 12 to 25. While few young people present to headspace with the specific aim of seeking treatment for an eating disorder, clinicians have observed that many young people present with some level of disordered eating. The aim of this study is to ascertain the prevalence of disordered eating in the young people, aged 15–25, attending headspace Camperdown in Inner Sydney. Young people presenting for assessment at headspace Camperdown are invited to complete a series of questionnaires online which includes selected items from the EDE-Q that assess for the presence of eating disorder symptoms occurring in the previous three-month period. Data collected from consenting young people will be analysed to determine the percentage of young people presenting to headspace Camperdown over a specific time period who self-reported experiencing eating disorder symptoms. Preliminary analyses indicate that over 30% of young people aged 15- 25 presenting to headspace Camperdown in a 6-month period self-reported as experiencing symptoms of disordered eating. Final results and conclusions are yet to be confirmed as analysis is currently ongoing. Practical considerations for the management of these symptoms within the context of a community youth mental health service will be discussed.

## H.3

### Exploring community perceptions of eating disorders and body image issues: How far have we actually come?

#### Kevin Barrow^1^

##### ^1^Butterfly Foundation, New South Wales, Australia

**Correspondence**: Kevin Barrow (kevin.barrow@thebutterflyfoundation.org.au)

*Journal of Eating Disorders* 2021, **9(1)**: H.3

Community understanding of mental health issues such as depression and anxiety has greatly improved. However, the same cannot be said for eating disorders. Although the efforts of the sector have helped to shift some perceptions, a significant level of misinformation and myth continue to perpetuate stigma and create barriers to help seeking, early intervention and treatment. Butterfly’s recent survey of over 3,000 people in Australia reveals how far we must go to change community perceptions of eating disorders and body image issues. The survey found that 1 in 4 people in Australia believe that eating disorders are a choice, and 1 in 4 perceive having an eating disorder to be a sign of weakness.

The inability of the community to recognise eating disorders is a critical barrier to service access. As a sector we must respond to these community attitudes and continue to be guided by the insights of people with lived experience in order to develop effective programs and services.

This presentation will highlight the major findings of Butterfly’s Community Insights Report, addressing existing perceptions about the prevalence, presentation and impacts as well as the drivers and barriers to engaging with services and support. Butterfly’s new framework which helps to ensure lived experience insights are at the centre of all Butterfly’s programs and services will also be shared.

## H.4

### Eating disorders: The lived experience carer peer mentor program

#### Belinda Chelius^1^, Emma Trappett^1^, Melissa Cheras^1^

##### ^1^Eating Disorders Queensland, Queensland, Australia

**Correspondence**: Belinda Chelius (admin@edq.org.au)

*Journal of Eating Disorders* 2021, **9(1)**: H.4

Eating Disorders Queensland (EDQ’s) Carer Peer Mentor Program (CPMP) is a six-month program where carers are matched with carer mentors for connection and support. The program follows a strengths-based model and recognises the value of a carer’s lived experience specific to eating disorders and the knowledge and wisdom developed as a result of their own caring role. The program is funded by QLD Health and has recently commenced its third round. It is recognised that caring for a loved one with an eating disorder can have impacts on carer wellbeing. Carer peer mentoring has been identified as beneficial for improving carer wellbeing, and the carer’s capacity to support their loved one (Visa and Harvey, 2017). In 2019, the CPMP program was piloted and evaluated by EDQ and the Queensland Eating Disorder Service (QuEDS) with key outcomes including increased hope, increased social support and carers feeling better informed to support their loved ones. Learnings and feedback from carers involved in the pilot have informed the adaptations to the current CPMP program. EDQ has continued to evaluate the benefits of the program using quantitative and qualitative data, with preliminary qualitative analysis demonstrating success of the program. This presentation will discuss the key elements which have contributed to the success of the CPMP program and will focus on learnings gained through carers’

## H.5

### The power of lived experience peer support and education for carers and the whole family

#### Violeta Bozinovski^1^, Christine Naismith^1^

##### ^1^Eating Disorders Families Australia, Victoria, Australia

**Correspondence**: Violeta Bozinovski (violeta.bozinovski@edfa.org.au)

*Journal of Eating Disorders* 2021, **9(1)**: H.5

Eating disorders are serious mental illnesses that not only have significant detrimental effects on the individual, but also affect the whole family. Research has shown the carers and family members of people with an eating disorder require support for their own mental health, as well as increased information about eating disorders.

This presentation will describe the model of peer support for carers and families designed by Eating Disorders Families Australia (EDFA) and an overview of the power of lived experience support and education for parents, carers and siblings of people who have an eating disorder will be provided We will also discuss the journey to creating a national lived-experience carer community and the rollout of the national ‘EDFA strive Carer Support groups.

In addition, we will also detail the lived experience EDFA Carer Education program and the EDFA Siblings Support groups.

Feedback on the programs will be discussed, as well as preliminary outcomes that describe the benefits of these programs, as well as the areas that require further attention.

## I.1

### Prospective and indirect relationships between social media use and body satisfaction among adolescents

#### Hannah Jarman^1^, Mathew Marques^1^, Siân McLean^1^, Amy Slater^2^, Susan Paxton^1^

##### ^1^La Trobe University, Victoria, Australia. ^2^The University of the West of England, Bristol, United Kingdom

**Correspondence**: Hannah Jarman (h.jarman@latrobe.edu.au)

*Journal of Eating Disorders* 2021, **9(1)**: I.1

Cross-sectional evidence suggests a small, negative association between social media use and body satisfaction. However, less is known regarding the prospective, reciprocal or mediating effects within this relationship. The current study used a three-wave design to examine the temporal relationships between social media use, thin-ideal and muscular-ideal internalisation, comparisons and body satisfaction. An additional aim was to inform theoretical models, whereby two operationalisations of social media use were examined in separate models (frequency vs photo-based use). Adolescents aged 11–16 years (N = 1,911, Mage = 14.27, SD = 1.08) completed three online surveys over 1-year. A series of cross-lagged panel models indicated acceptable fit for both operationalisations of social media use, with better fit statistics for the frequency rather than photo-based model. For both models, higher social media use predicted higher thin-ideal internalisation and comparisons, which in turn predicted lower body satisfaction. In the reciprocal direction, lower body satisfaction predicted higher thin-ideal internalisation and comparisons, which in turn predicted higher social media use. These effects were inconsistent across models. Finally, both models demonstrated gender equivalence, suggesting that intervention efforts aimed at reducing mediators and their effects on body image may be suitable for both boys and girls. These finding provide prospective support for the indirect relationships between social media use and body satisfaction and have implications for sociocultural theory.

## J.1

### Case series evaluation of an early intervention program for eating disorders in low socio-economic status populations

#### Marcela Radunz^1^, Luke Pritchard^2,3^, Eloisa Steen^4^, Paul Williamson^1^, Tracey Wade^1^

##### ^1^Flinders University, South Australia, Australia. ^2^Sonder/emerge, South Australia, Australia. ^3^Advanced Psychology Services, South Australia, Australia. ^4^Sonder Edinburgh North, South Australia, Australia

**Correspondence**: Marcela Radunz (marcela.radunz@flinders.edu.au)

*Journal of Eating Disorders* 2021, **9(1)**: J.1

Eating disorders (EDs) are serious mental illnesses, with anorexia nervosa having the highest mortality rate of any mental illness, making the need for early intervention crucial. The ED field has been much slower to embrace the concept of early intervention, with much of the literature focusing on prevention and treatment, with very little research investigating early intervention strategies and programs, particularly in populations of low-socio-economic status (SES). Informed by the original model and findings from the FREED study (McClelland et al., 2018), an early intervention service for EDs in a primary health care setting in South Australia was established in two low SES areas. Emerge for Eating Disorders (emerge- ED) aims to provide treatment as early as possible to those experiencing initial symptoms of disordered eating, focusing on the promotion of early help-seeking. The present study provides a pilot evaluation of these services, assessing ED and other clinical outcomes in this treatment seeking sample over time. Linear Mixed Model analyses were performed to evaluate within group changes in clinical outcomes for the emerge-ED sample over the course of treatment. Significant main effects for time were found across all treatment outcomes measures, with participants showing large within-group effect size decrease from baseline to end of treatment. Multilevel Modelling was performed to evaluate changes over time in a disordered eating session-by-session measure.

## J.2

### Binge eating e-therapy: Translating evidence into mainstream practice

#### Sarah Barakat^1^, Stephen Touyz^1^, Danielle Maloney^1^, Janice Russell^1^, Phillipa Hay^2^, Michelle Cunich^3^, Ang Li^3^, Sloane Madden^4,5,6^, Jane Miskovic-Wheatley^7^, Sarah Maguire^7^

##### ^1^University of Sydney, New South Wales, Australia. ^2^University of Western Sydney, New South Wales, Australia. ^3^Boden Institute, University of Sydney, New South Wales, Australia. ^4^The Sydney Children’s Hospital Network, New South Wales, Australia. ^5^The Redleaf Practice, New South Wales, Australia. ^6^Westmead, New South Wales, Australia. ^7^InsideOut Institute, University of Sydney, New South Wales, Australia

**Correspondence**: Sarah Barakat (sarah.barakat@sydney.edu.au)

*Journal of Eating Disorders* 2021, **9(1)**: J.2

Cognitive behaviour therapy (CBT) is the treatment with the strongest evidence for bulimia nervosa (BN), however access to skilled clinicians and the cost of treatment are major barriers to receiving access to care. Binge Eating eTherapy (BEeT) is an online CBT program, which aims to overcome several barriers associated with face-to-face delivery of treatment. BEeT consists of ten one-hour interactive, multi-media sessions and includes an inbuilt calendar with several self-monitoring tools. The current study aims to investigate the effectiveness of BEeT in bringing about a reduction in bulimic symptomology in a sample of individuals with BN. The presentation will provide an update on a randomised controlled trial (RCT) which is currently in progress and aims to compare the effectiveness of engaging in the BEeT program in a purely independent manner to use of BEeT in conjunction with regular support from a non-specialist to waitlist control. Supported BEeT will be examined across several health settings to explore site-specific outcomes of implementation. The presentation will provide an update on the findings of the RCT, as well as outline the findings from a 4-week pilot of BEeT involving 25 females with BN, which found significant improvements in several outcomes including objective binge episodes. Conference attendees will gain insight into the ways in which evidence-based online treatments can be embedded within a stepped-care model.

## P.1

### Therapeutic interventions for eating disorders as an alternative to the medical model

#### Jess Tone^1^, Belinda Chelius^1^

##### ^1^Eating Disorders Queensland, Queensland, Australia

**Correspondence**: Belinda Chelius (belindac@edq.org.au)

*Journal of Eating Disorders* 2021, **9(1)**: P.1

Eating Disorders Queensland (EDQ) is a not-for-profit organisation funded by Queensland Health to provide the largest community eating disorder treatment services for individuals and their families in the state. EDQ treatment services are centred within a social justice framework and hold a strong feminist lens. EDQ utilises a trauma-informed approach allowing the client to tell their story. At EDQ, eating disorders are not viewed as an individual pathology, instead as a broader phenomenological presentation. In treatment the social, environmental, and political impacts of an eating disorder is explored. The aim of this paper will be to look at the data and client stories to ascertain what was experienced and how was it experienced, using a phenomenological approach.

Within individual counselling, EDQ therapists utilise a multidisciplinary approach to eating disorders, individually tailoring of treatment programs, with the efficacy of the person-centred approach rooted in the therapeutic alliance (Berg, 2018) modelling a collaborative approach, educating on these systems and being led by individual choice. This is completed alongside clinical management to ensure the medical issues that coincide with the eating disorder are managed.

## P.2

### A positive evolution: How covid changed the EDFA strive carer support groups from in-person to online

#### Helen Searle^1^, Christine Naismith^1^

##### ^1^Alfred Health, Victoria, Australia

**Correspondence**: Helen Searle (helendsearle@gmail.com)

*Journal of Eating Disorders* 2021, **9(1)**: P.2

Eating Disorders Families Australia (EDFA) commenced the first in-person strive (Support, Teach, Reassure, Inform, Validate, Empower) carer support group in Melbourne in 2016 and by 2019 had two more in WA and Bendigo. Setting up additional groups was time and resource intensive. Covid created an opportunity to pivot to monthly online groups run by trained volunteer carer facilitators. The purpose of this study was to assess whether we could provide access to peer support more widely, to more people, with qualitative data feedback on the benefits of meeting via zoom. Facilitators were recruited and trained online via zoom, and monthly groups were launched and conducted in every state and territory in Australia. A poll at the conclusion of each meeting allowed measurement of qualitative aspects of satisfaction and connection. This research demonstrated that zoom allowed us to reach more carers nationally, that carers appreciated the opportunity to connect and meet in this way, but that the numbers of rural attendees did not increase as expected. Future research will trial additional methods to reach and involve carers in regional and rural locations.

## P.3

### Outcomes of a regional day program treatment service for eating disorders

#### Emma Gallagher^1^, James Fletcher^1^

##### ^1^Hospital Eating Disorders Day Program, New South Wales, Australia

**Correspondence**: Emma Gallagher (emma.gallagher1@health.nsw.gov.au)

*Journal of Eating Disorders* 2021, **9(1)**: P.3

The Eating Disorders Day Program in Newcastle supports clients with Anorexia Nervosa, Bulimia Nervosa and Binge Eating Disorder. It is a semi-closed group format running for up to 12 weeks, for up to eight clients at a time. Clients receive meal support for the provided breakfasts, morning teas and lunches, as well as CBT-E and DBT group therapy. The program also has a strong focus on sensory processing and mental health rehabilitation.

In addition to weekly weighing, clients regularly complete measures of eating disorder symptomatology (EDEQ), body image, emotion regulation, quality of life, self-compassion, valued living, and depression anxiety and stress. The poster will outline client feedback regarding their experiences of the program, as well as the effectiveness of the program for each clinical group at discharge. Key effectiveness markers include weight change, Global EDEQ score, and quality of life domains. Correlational outcomes will be presented for the last five years, highlighting which variables are linked to positive outcomes in weight restoration and changes in eating disorder symptomatology.

## P.4

### Family based treatment via telehealth for rural and regional adolescents with anorexia nervosa: Protocol and preliminary findings

#### Ashlea Hambleton^1^

##### ^1^University of Sydney, New South Wales, Australia

**Correspondence**: Ashlea Hambleton (ashlea.hambleton@hotmail.com)

*Journal of Eating Disorders* 2021, **9(1)**: P.4

Family-based treatment (FBT) is an efficacious outpatient intervention for young people diagnosed with Anorexia Nervosa (AN). To date, treatment to protocol has relied on standard face-to-face delivery, which is subject to geographic, temporal and human factors, rendering it susceptible to inequities and disruption, resulting in poorer service provision for rural families. The COVID-19 pandemic has created an unprecedented demand for telemedicine to facilitate the continuity of care, rendering the urgent evaluation and optimisation of providing evidence-based therapies via this modality critically important. The primary aim of the current study was to examine whether an unspecialised rural workforce and service model can be safely and effectively translated to digitally deliver FBT for AN to address these access issues. Thirty young people aged 12 to 18 years who meet DSM-5 diagnostic criteria for AN/Atypical AN, and live in a rural or regional setting, along with their family, are being recruited to the study. Therapists will provide 18 sessions of FBT over nine months via telemedicine, directly into the family’s home. Initial quantitative (%mBMI and EDE) and qualitative (acceptability and feasibility) findings from the first wave of families will be presented. If delivering treatment in this modality is clinically effective, economically feasible, and health services and workforce can adapt to this alternative model, currently existing access inequalities will be minimised.

## P.5

### Examining negative emotions for bodily changes associated with pregnancy in young Japanese women

#### Yuko Yamamiya^1^, Mika Omori^1^

##### ^1^Ochanomizu University, Tokyo, Japan

**Correspondence**: Yuko Yamamiya (yuko.yamamiya@gmail.com)

*Journal of Eating Disorders* 2021, **9(1)**: P.5

Young women who desire thinness while avoiding fatness may see the increased body size and weight occurring during pregnancy negatively. A total of 200 Japanese young women who had never become pregnant filled out questionnaires. As the scale named Negativity toward Bodily Changes during Pregnancy Scale that assesses negative emotions toward the changes in overall appearance, weight/shape, and breasts during pregnancy was developed and validated on pregnant women, its psychometric properties were established with the current sample. Correlation analyses showed that those with high thin-ideal internalisation, body dissatisfaction, and drive for thinness tended to feel negatively about the changes in overall appearance, weight/shape, and breasts.

Moreover, high appearance comparison and low ideal BMI were associated with negative emotions for all changes, except the change in breasts. On the contrary, self-reported current BMI was positively correlated with negativity toward the change in breasts only. Lastly, regression analyses indicated that thin-ideal internalisation was the predictor of all changes, whereas appearance comparison, body dissatisfaction, ideal BMI, and drive for thinness predicted one of the changes along with thin-ideal internalisation. As thin-ideal internalisation and drive for thinness seem to predict the negative emotions in both non-pregnant and pregnant women, targeting them is thought to prevent negative emotions that pregnant women often experience when their body progressively changes.

## P.6

### Orthorexia nervosa in women with a current or past eating disorder

#### Stephanie Miles^1^, Maja Nedeljkovic^1^, Andrea Phillipou^1, 2, 3, 4^

##### ^1^Centre for Mental Health, Swinburne University of Technology, Melbourne, Australia. ^2^Department of Mental Health, St Vincent’s Hospital, Melbourne, Australia. ^3^Department of Psychiatry, The University of Melbourne, Melbourne, Australia. ^4^Department of Mental Health, Austin Health, Melbourne, Australia

**Correspondence**: Stephanie Miles (smiles@swin.edu.au)

*Journal of Eating Disorders* 2021, **9(1)**: P.6

Orthorexia nervosa (ON) refers to a fixation with ‘healthy’ eating and high-quality food, and has been proposed as an eating disorder (ED). However, there has been considerable debate in the literature if ON is a unique psychiatric disorder, or a symptom of other EDs. This study aimed to explore symptoms of ON in people with a lifetime ED and people with no history of an ED (controls). Thirty-five women with current anorexia nervosa (AN), 27 women with a past diagnosis of AN and 171 controls completed the ORTO-7 (a self-report questionnaire of ON severity) as part of an online study (age M = 28.75, SD = 9.42). Women with current AN scored significantly lower on the ORTO-7 (indicating greater ON severity) than women with past AN and controls. There were no significant differences between women with past AN and the controls in ON symptom severity. After examining cut-off scores, it was found that 94% of women with current AN met the cut-off score for probable ON, whilst 59% of women with past AN and 50% of controls met the cut-off score. These findings indicate that although ON may co-occur with other EDs, ON may be a standalone psychiatric disorder which can occur in people with no history of an ED.

## P.7

### Understanding the development of pathological dieting: A longitudinal psycho-biological study of dieting in young people

#### Mirei Okada^1^

##### ^1^The University of Sydney, New South Wales, Australia

**Correspondence**: Mirei Okada (mirei.okada@sydney.edu.au)

*Journal of Eating Disorders* 2021, **9(1)**: P.7

Underlying mechanisms involved in the onset and maintenance of the most life-threatening eating disorder, Anorexia Nervosa, are poorly understood. We seek to address the significant gap in knowledge by conducting a longitudinal study of the highest risk age group, young people aged 16–25, engaging in dieting to assess psychological and physiological responses to dieting, alongside self-reported feeding behaviour, thereby tracking the natural pathophysiology of dieting. Our aim is to determine and identify changes in underlying biological and psychological risks (neuro, endocrine, microbes, physiology) in young individuals dieting and determine which of these risks lead to pathological dieting and the possible onset of an eating disorder. Females and males aged 16–25 (n = 500) who are engaging in self-initiated dieting will be recruited to complete an assessment of eating disorder symptoms, general mental health and food intake, along with other measures of hunger, satiety, gastrointestinal movements and diet compliance. These assessments will be completed monthly over a 6-month period. A targeted embedded subset (n = 50) will have an additional detailed characterisation of physiological markers known to vary with diet history (via gut microbiome), eating patterns (via endocrine hormones and metabolites) and genetic material. This will be collected through stool/faeces, blood and saliva specimens at start-middle-end points. The current study is on-going and methodologies will be presented.

## P.8

### The experience of adults recovering from an eating disorder in professionally-led support groups

#### Archana Waller^1^

##### ^1^Australian College of Applied Psychology, New South Wales, Australia

**Correspondence**: Archana Waller (archana.waller@gmail.com)

*Journal of Eating Disorders* 2021, **9(1)**: P.8

The study explored the experience of adults recovering from an eating disorder in a professionally-led monthly support group. Participants were 18 adults recovering from an eating disorder who attended a monthly support group. The aim was to explore the experience of eating disorder support group participants through a qualitative approach. The data were collected using an online anonymous interview and then analysed using a thematic analysis. The main themes that emerged were: (1) sharing the pain and promise, (2) cautions and concerns and (3) facilitators have influence. The findings indicate that the support group provided a safe space to share their lived experience, that it reduced stigma and isolation, and improved participants’ motivation and engagement. Moreover, the results revealed some challenges to the functioning of the group. These included management of discussions and dominant members, need for psycho-educational information and managing intense feelings, relating to body-related comparison and other mental disorder comorbidities. This is the first study highlighting the valuable role of the facilitator in balancing content with compassion, in ensuring safety in the group, and potentially fulfilling a valuable education function in supporting participants in their eating disorder recovery journey.

## P.9

### Changes in urine pH during the nutritional rehabilitation of adolescent and young adult patients hospitalised with a restrictive eating disorder

#### Elizabeth Parker^1^

##### ^1^Westmead Hospital, New South Wales, Australia

**Correspondence**: Elizabeth Parker (Elizabeth.Parker@health.nsw.gov.au)

*Journal of Eating Disorders* 2021, **9(1)**: P.9

**Aims:** Patients with restrictive eating disorders (EDs) typically have alkaline urine (pH ≥ 7) on presentation. This study investigates the change of urine pH in adolescent and young adult patients hospitalised with restrictive EDs.

**Methods.** Retrospective chart review of patients (aged 14–25) admitted for ≥ 14 days during 2018–2019. Data were analysed using linear mixed effects models to investigate change in urine pH and associations with: degree of malnutrition, medical instability and purging behaviours.

**Results.** 79 patients (95% female) with mean age 17.7 ± 1.4 years were included. A significant decrease in patients with urine pH ≥ 7 was observed from day 1 (baseline) and after 1 week of refeeding (56.6% vs. 29.9%, p = 0.001). Urine pH decreased from mean 7.0 ± 0.9 at baseline to 6.6 ± 0.7 after 1 week of refeeding. Patients admitted with medical instability, a lower percentage median body mass index, and without purging behaviours were more likely to present with a urine pH ≥ 7. Conclusion. The likelihood of an alkaline urine was greatest in those most malnourished. A change in urine pH from ≥ 7 to < 7 was observed after 1 week of nutritional rehabilitation. Further research is required to examine the underlying mechanisms causing the elevation of urine pH, and the change that occurs during refeeding.

## P.10

### The COVID-19 pandemic: Psychological and behavioral responses to the shutdown of the beauty industry

#### Toni Pikoos^1^

##### ^1^Swinburne University of Technology, New South Wales, Australia

**Correspondence**: Toni Pikoos (tonipikoos@gmail.com)

*Journal of Eating Disorders* 2021, **9(1)**: P.10

**Background:** During COVID-19 ‘lockdowns’, Australia implemented widespread closure of beauty and cosmetic services. The current study explored the relationship between engagement in appearance- focused behaviors and distress regarding beauty service closure. Participants with high and low levels of dysmorphic concern were compared to determine whether COVID-19 restrictions may affect these groups differently.

**Method:** 216 participants living in Australia in May 2020 completed an online survey. Questions addressed engagement in appearance-focused behaviors during the COVID-19 pandemic and attitudes towards beauty service closure. The Dysmorphic Concern Questionnaire (DCQ) was used to group participants by low and high dysmorphic concern.

**Results:** Appearance-focused behaviors decreased in the low DCQ group (n = 163) during the COVID-19 pandemic, while such behaviors in the high DCQ group (n = 53) remained unchanged. Individuals who were living alone, younger, reported higher dysmorphic concern and greater distress over beauty service closure engaged in more frequent appearance-focused behaviors. The high DCQ group reported greater distress over beauty service closure, and increased desire to obtain future beauty treatments.

**Discussion:** While COVID-19 restrictions may have provided a break from societal appearance pressure for those with low dysmorphic concern, appearance- focused behaviors persisted in individuals with high dysmorphic concern. These findings illustrate the potential for COVID-19 lockdowns to exacerbate appearance-related distress, and contribute to knowledge regarding the mental health effects of the COVID-19 pandemic.

## P.11

### Investigating the effects of self-monitoring through ecological momentary assessment in obesity related eating behaviours: A pilot study

#### Monique Shipp^1^, Jayanthi Raman^2^,  Sylvie Berthoz^3^, Shahin Rahimi-Golkhandan^4^, Joel Swendsen^5^, Pierre Schweitzer^6^

##### ^1^Australian College of Applied Psychology, New South Wales, Australia. ^2^University of Technology Sydney, New South Wales, Australia. ^3^French National Institute of Health & Medical Research, Bordeaux, France. ^4^Australian College of Applied Psychology, New South Wales, Australia. ^5^Director of Research- Bordeaux Neurocampus, Bordeaux, France. ^6^National Centre for Scientific Research (CNRS - UMR 5287), Bordeaux, France

**Correspondence**: Monique Shipp (moniqueshipp@gmail.com)

*Journal of Eating Disorders* 2021, **9(1)**: P.11

This study investigated the effects of incidental self-monitoring in individuals with obesity when participants completed an ecological momentary assessment (EMA) study. Data on disordered eating behaviours (DEB) were collected in NSW, Australia during the COVID-19 period with pandemic restrictions of “essential travel only”. We also present self-reported qualitative data to elucidate the impact of the COVID 19 pandemic on the eating behaviour of our participants during this time. Over 14 days, Australian adults (n = 8; Age range = 37.88 years) received four randomly prompted EMA questionnaires on a smartphone.

Self-reported pre-post data was obtained on the following variables: emotional eating, addictive eating, impulsivity, emotion regulation, cognitive flexibility, depression, anxiety and stress. Findings showed statistically meaningful changes in addictive eating symptoms. Further, self-report data suggested the observed changes may have been impacted by the ongoing COVID-19 pandemic. This study also provides insight into disordered eating behaviour and food/drink shopping habits in the context of COVID-19 pandemic crisis for individuals with a BMI of > 30. Future research should further explore the effects of self-monitoring impacting the daily determinants of DEB.

## P.12

### Reflections from wise choices group program at Eating Disorder Service Sunshine Coast (EDS-SC)

#### Copeland Winten^1^

##### ^1^QLD Health, Queensland, Australia

**Correspondence**: Copeland Winten (copeland.winten@gmail.com)

*Journal of Eating Disorders* 2021, **9(1)**: P.12

Wise Choices is an Acceptance and Commitment Therapy ten-week group program run through the Eating Disorder Service- Sunshine Coast. EDS-SC identified a need for the program due to comorbid presentations of personality disorders/traits in consumers that may have been impeding access to treatment. It has been run three times as a therapy stream in the service, with a fourth group upcoming. Through this time consumers and clinicians have provided feedback and reviewed outcomes to improve the program to be more specific to disordered eating/eating disorders. Participants have come from all genders, ages, diagnoses and various stages of treatment and recovery. Attendance varied and was related to diagnoses; a restrictive dietary pattern was related to poorer outcomes and attendance. Consumer feedback has been significantly positive. The largest variation to the original program from consumer feedback is the addition of nutrition and psychoeducation segment. This allowed facilitated discussion around specific eating disorder topics. Variations to the original Wise Choices model, considerations for eating disorders, outcomes, attendees and clinician feedback will be discussed and reviewed in this session.

## P.13

### Body image, disordered eating and compulsive exercise in women who post different types of material on Instagram

#### Phoebe Yu Wu^1^, Jane Harford^1^, Ivanka Prichard^1^

##### ^1^Flinders University, South Australia, Australia

**Correspondence**: Phoebe Yu Wu (phoebe.wu@flinders.edu.au)

*Journal of Eating Disorders* 2021, **9(1)**: P.13

**Introduction:** Substantial research has demonstrated a link between social media use and body image concerns. In particular, different forms of popular idealised imagery (e.g., fitspiration, clean eating, fashion) promote unattainable ideals and have been linked to negative body image and unhealthy behaviours among women. The aim of the present study was to examine the relationship between posting appearance-focused, wellness-focused, and other forms of imagery on women’s body image, disordered eating, and compulsive exercise.

**Method:** Participants 304 females aged between 18 and 55 years who posted material to a range of different hashtags on Instagram completed an online questionnaire.

**Results:** Women who post imagery to appearance-focused hashtags (i.e., fitspiration, clean eating, fashion) had lower levels of body dissatisfaction than women who posted to other hashtags or did not use hashtags. Women who posted to wellness-focused hashtags (i.e., fitspiration and clean eating) had higher levels of compulsive exercise and athletic-ideal internalisation than women in the other groups. Appearance-ideal internalisation did not moderate these relationships.

**Conclusion:** Overall, the findings of this study suggest that posting certain types of imagery on Instagram may not be linked to negative body image as strongly as the exposure to similar content does. Nonetheless, further investigation is needed to construct a comprehensive understanding of the characteristics of women who post certain types of imagery on social media.

**Keywords: **social media, disordered eating, compulsive exercise

